# Biochemical Profiles, DPPH Radical-Scavenging Activity, and Associated Soil Properties of Wild *Galium verum* L. and *Galium aparine* L. Populations in Southeastern Bulgaria

**DOI:** 10.3390/plants15192917

**Published:** 2026-09-23

**Authors:** Margarita Koleva Petkova, Mima Hristova Todorova, Milena Tankova Tzanova, Neli Hristova Grozeva

**Affiliations:** 1 Department of Biological Sciences, Faculty of Agriculture, Trakia University, 6000 Stara Zagora, Bulgaria; milena.tsanova@trakia-uni.bg (M.T.T.); n.grozeva@trakia-uni.bg (N.H.G.); 2 Department of Plant Breeding, Faculty of Agriculture, Trakia University, 6000 Stara Zagora, Bulgaria; mima.todorova@trakia-uni.bg

**Keywords:** *Galium*, DPPH radical-scavenging activity, total phenolic content, total flavonoid content, edaphic properties, Spearman’s rank correlation, principal component analysis, soil–plant interactions, Bulgaria

## Abstract

Biochemical variation in wild medicinal plants may reflect both species-associated characteristics and habitat conditions. This study characterized six wild populations (three per species) of *Galium verum* L. and *Galium aparine* L. from southeastern Bulgaria using matched soil–plant sampling. Plant material was analyzed for proximate composition, total phenolic content (TPC), total flavonoid content (TFC), anthocyanin assay responses, and 2,2-diphenyl-1-picrylhydrazyl (DPPH) radical-scavenging activity; associated soils were characterized for physicochemical properties, nutrient concentrations, and texture. Descriptive species-associated summaries showed that *G. verum* had higher mean DPPH values (26.4 vs. 14.7 µmol Trolox equivalents per gram of dry extract), percentage inhibition (49.0% vs. 21.3%), and slightly higher TFC. Conversely, *G. aparine* had higher mean crude protein, ash, and TPC. The anthocyanin assay produced only trace-level responses, which were generally higher in *G. aparine* and were interpreted qualitatively. Exploratory principal component analysis showed coordinated variation in plant and soil variables and separated the two species-associated groups along the first component. Two-sided exact permutation Spearman analysis at the sampling-point level (*n* = 6) identified nominal associations, including zinc (Zn) with crude protein (ρ = 1.000) and cation exchange capacity with TFC (ρ = −0.943). Because species identity coincided with sampling site and collection period, while aerial biomass composition was not quantified, the observed patterns cannot distinguish independent taxonomic, seasonal, and edaphic effects or support causal inference. By providing ecological context for the biochemical heterogeneity of Bulgarian populations of *G. verum* and *G. aparine*, this integrated soil–plant approach offers a descriptive, hypothesis-generating basis for further phytochemical and ecological investigations using balanced sampling across comparable habitats and collection periods.

## 1. Introduction

The genus *Galium* L. (Rubiaceae) comprises a taxonomically diverse group of herbaceous species characterized by extensive geographical distribution, high ecological plasticity, and pronounced variation in habitat requirements. Recognized as the largest and one of the most widely distributed genera within the tribe Rubieae, *Galium* includes species adapted to a broad spectrum of environmental conditions, ranging from semi-natural ecosystems to highly disturbed habitats [1,2,3]. In particular, *G. aparine* has been documented from diverse lowland and disturbed habitats across Europe, whereas *G. verum* has been reported from mountains, grasslands, meadows, river beaches, open fields, ditch sides, streamsides, wet places, forests, thickets, and valleys over a broad elevational range extending from near sea level to 4100 m. This ecological diversity highlights the suitability of *Galium* for investigating associations between habitat-specific environmental conditions, plant nutritional status, and metabolic variability [4,5].

Among the species examined in the present study, *G. verum* and *G. aparine* differ in life history and habitat occupancy. *G. verum* is a perennial taxon occurring across diverse open and semi-open environments. *G. aparine* is an annual climbing or scrambling herb that often colonizes nutrient-enriched or disturbed sites and can exploit surrounding vegetation as physical support [5,6,7].

The phytochemical importance of *G. verum* has been emphasized in recent reviews summarizing its diverse bioactive constituents [8]. To complement these insights, foundational historical works and older screening literature have long established the basic qualitative chemical framework of the genus [9,10], while more recent investigations have confirmed the occurrence of flavonoids, phenolic acids, iridoids, anthraquinones, steroids, and other specialized metabolites in the genus, together with antioxidant and other biological activities [11,12]. A 2025 phytochemical study of *G. aparine* documented multiple compound classes of chemotaxonomic relevance, including flavonoids, phenolic acids, an iridoid, anthraquinones, nucleosides, and steroids [13]. Comparative studies involving *G. verum* and *G. aparine* have demonstrated species-related differences in phenolic composition, flavonoid abundance, and antioxidant potential [14]. These two species were therefore selected because their taxonomic relatedness, occurrence within the same broad floristic regions of Bulgaria, contrasting life histories and habitat affinities, and documented phytochemical relevance provide a focused framework for comparing their biochemical profiles in relation to associated soil properties.

Nevertheless, phytochemical diversity among natural populations is unlikely to be determined exclusively by genetic background, as environmental conditions may substantially modify metabolite accumulation within and among species. Increasing evidence from medicinal plant research indicates that plants growing under different light regimes, moisture conditions, soil reaction, and nutrient supply may express substantially altered biochemical profiles, even when they belong to the same species [15].

In this context, edaphic factors are especially important because they simultaneously influence mineral nutrition, osmotic balance, rhizosphere interactions, and enzymatic processes linked to primary and secondary metabolism. Soil pH affects nutrient solubility and availability [16,17], whereas phosphorus (P) is central to phosphate homeostasis and energy-related metabolism [18,19]. Sulfur (S) is integral to core metabolic processes and stress-related biochemical functions [20]. Zinc (Zn) and copper (Cu) are essential micronutrients that serve as structural or catalytic cofactors in numerous proteins and enzymes involved in plant metabolism and stress responses [21,22,23]. Soil organic matter (OM) contributes to nutrient retention, water-holding capacity, aggregate stability, and microbial activity [24,25]. The contrasting ecological strategies of *G. verum* and *G. aparine* may be associated with differences in nutrient acquisition, responses to environmental stress, and specialized metabolite profiles [15,17,26,27]. Therefore, an integrated analysis of plant and soil composition can support a more comprehensive interpretation of naturally occurring biochemical variability [15,26].

Previous phytochemical studies of *G. verum* and *G. aparine* have reported radical-scavenging or antioxidant responses of their extracts [8,14]. In the present study, the 2,2-diphenyl-1-picrylhydrazyl (DPPH) assay was selected as a comparative in vitro screening measure of the radical-scavenging activity of crude methanolic extracts. Because it represents a single chemical assay, it was not intended to provide a comprehensive assessment of antioxidant capacity or to demonstrate biological efficacy or a specific practical application. Environmental factors, including soil properties, climatic conditions, and abiotic stress, can alter the accumulation of secondary metabolites and the biochemical characteristics of plant material [15,26,27,28,29]. Consequently, the absence of corresponding soil characterization may limit the ecological interpretation of biochemical differences associated with species identity, environmental variation, or both [30].

The present study integrates plant and soil analyses from six defined sampling points in Bulgaria. One population of each species was sampled in each of the Eastern Stara Planina, Thracian Lowland, and Tundzha Hilly Plain floristic regions. These sites represent distinct ecological contexts, including open field margins, river-associated habitats, shrub-dominated habitats, and soils differing in texture, nutrient availability, and pH. This sampling framework allowed biochemical and edaphic variation to be described across the six sampling points within a geographically constrained but environmentally heterogeneous study area. However, it was not designed to separate the independent effects of species identity, sampling site, collection period, and associated soil conditions.

Based on the ecological and edaphic contrasts among the studied sites, we hypothesized that the sampled populations of *G. verum* and *G. aparine* would exhibit differing biochemical profiles and that site-specific soil characteristics, particularly pH and micronutrient availability, would be associated with variation in DPPH radical-scavenging activity and proximate composition.

Accordingly, the aim of the present study was to characterize the proximate composition, phytochemical content, and DPPH radical-scavenging activity of six wild populations of *G. verum and G. aparine* from southeastern Bulgaria and to examine these traits in relation to the associated soil properties. By linking plant chemical profiles with environmental measurements, this study provides ecological context for biochemical variability in *G. verum* and *G. aparine* under natural field conditions.

The specific objectives were: (i) to summarize the analytical profiles of each sampling point; (ii) to provide a descriptive comparison of the mean compositional and phytochemical characteristics and DPPH radical-scavenging activity of the *G. verum* and *G. aparine* samples; (iii) to describe the chemical and textural properties of the soils associated with each sampling point; and (iv) to explore multivariate patterns and site-level soil–plant associations using principal component analysis (PCA) and exact-permutation Spearman’s rank correlation analysis. These objectives provide a framework for describing biochemical and edaphic variation among the six sampled populations and for generating hypotheses to be tested in larger, balanced studies.

## 2. Materials and Methods

### 2.1. Study Area and Plant Material

Wild populations of *G. verum* L. and *G. aparine* L. were collected from six distinct sampling points located within three floristic regions of Bulgaria: the Eastern Stara Planina, Thracian Lowland, and Tundzha Hilly Plain regions. Three populations were sampled for each species, with each population representing an independent field location characterized by specific ecological and edaphic conditions. The selected sites represented contrasting habitats, including open field margins, river-associated habitats, and shrub-dominated vegetation, and differed in soil texture, nutrient availability, and soil reaction. The geographic location, altitude, and floristic affiliation of the six sampling points (P1-P6) are presented in Table 1. The corresponding sampling-site data are also provided in Supplementary Data S1.

Aerial plant parts were collected at full flowering on 9 May 2025 at P4, 17 May 2025 at P5, and 18 May 2025 at P6 for *G. aparine*, and on 16 June 2025 at P1, 27 June 2025 at P2, and 29 June 2025 at P3 for *G. verum*. The difference in collection dates reflected differences in the timing of full flowering between the two species under local field conditions. Consequently, the collection period was completely aligned with species identity, and potential seasonal influences could not be distinguished from the biochemical differences between the two groups of samples. The collected plant material consisted of apical shoot portions approximately 30–40 cm long, which were cut with scissors above ground level so that the roots were excluded. The samples included stems bearing leaves and reproductive structures (flowers and, where present, developing fruits). Although sampling was standardized by the approximate length of the collected shoot portions, the relative proportions of stems, leaves, flowers, and fruits were not separately quantified. Plant identification was performed using standard taxonomic keys and authoritative floristic references for the Bulgarian flora [31,32]. Following collection, the plant material was transported to the laboratory and processed as described in Section 2.4.

### 2.2. Soil Sampling

Root-zone soil samples associated with each studied plant population were collected during the same field campaign as the corresponding plant material to ensure direct soil–plant correspondence. At each site, soil material was collected from a depth of 0–30 cm using a soil probe. Sampling followed the general representative-sampling principles recommended by Lancrop Laboratories (Pocklington, UK), including transect-based collection and preparation of a composite sample from multiple subsamples. For each population, 15–20 individual cores (subsamples) were collected along a zigzag transect, depending on the spatial extent of the plant population. The subsamples were then pooled, thoroughly mixed, and homogenized to obtain one representative composite sample per site. The final mass of each composite soil sample ranged from 600 to 800 g. Before dispatch to the laboratory, the samples were air-dried, cleaned of stones and plant debris, gently crushed, double-bagged, labeled, and transported by express courier to Lancrop Laboratories for analysis.

### 2.3. Soil Analysis

Soil analyses were performed by Lancrop Laboratories (Pocklington, UK), a laboratory holding United Kingdom Accreditation Service (UKAS) accreditation for specified testing activities. The composite soil samples were air-dried before analysis, as described in Section 2.2. The analytical dataset included soil pH, organic matter, cation exchange capacity (CEC), plant-available macroelements and microelements, and particle-size distribution, including sand, silt, and clay fractions.

Soil pH was determined using the Macauley method, with calcium chloride (CaCl_2_) as the extractant. Soil organic matter was determined by loss on ignition (LOI) at 500 °C. Plant-available phosphorus was extracted using the Olsen method with sodium hydrogen carbonate and determined by discrete colorimetric analysis following reaction with ammonium molybdate. Plant-available sulfur was extracted using calcium tetrahydrogen diorthophosphate and determined by inductively coupled plasma (ICP) analysis. Plant-available copper and zinc were extracted with 0.05 M disodium ethylenediaminetetraacetate (EDTA) and determined by ICP analysis. Cation exchange capacity was determined after rinsing the soil with water and subsequent leaching with 1 M ammonium acetate, followed by ICP analysis.

Particle-size distribution was determined by Lancrop Laboratories as part of its soil-texture analysis service and was reported as percentages of sand, silt, and clay, together with the corresponding soil-texture classification. The specific documented in-house procedure used for particle-size determination was not provided in the analytical report or in the publicly available laboratory documentation.

These variables were used to characterize soil fertility, nutrient availability, and textural class at each sampling point, and subsequently to explore their potential associations with the biochemical traits of the studied *Galium* populations.

### 2.4. Preparation of Plant Material

The harvested aerial plant material was manually cleaned without washing to remove visible soil particles and foreign material. Samples from both species were then dried for five days under identical ambient laboratory conditions (18–20 °C). The material was spread in a thin layer in a dark, well-ventilated area and turned daily to promote uniform drying. The same drying procedure was applied to both species, despite their collection in different months. After drying, the material was ground to a fine homogeneous powder (particle size < 400 µm) using a laboratory mill. The powdered samples were stored under dry, light-protected conditions until further analysis. Standardized powdered material was subsequently used for proximate analyses and ultrasound-assisted methanolic extraction.

### 2.5. Preparation of Methanolic Extracts and Determination of Dry Extract

Methanolic extracts were prepared from the dried and powdered aerial parts of the studied *Galium* species using 99.9% methanol at a ratio of 1 g plant material to 10 mL solvent. Ultrasound-assisted extraction was performed for 30 min at room temperature using an Elmasonic S30 ultrasonic bath (Elma Schmidbauer GmbH, Singen, Germany) operating at a frequency of 37 kHz and a nominal effective ultrasonic power of 80 W. The resulting extracts were filtered through laboratory-grade filter paper to remove suspended plant particles.

The dry-extract concentration of each methanolic extract was determined gravimetrically by evaporating a 0.5 mL aliquot to dryness and calculating the mass of the resulting residue per milliliter of extract. Dry-extract yield was expressed as a percentage of the initial dry plant mass.

Based on the gravimetrically determined concentration, a working solution containing 1.0 mg dry extract/mL was prepared from each extract by dilution with methanol. For each sampling period, the working solutions were subsequently used for the phytochemical and DPPH radical-scavenging analyses on the day of preparation, without prolonged storage. The quantitative spectrophotometric results were calculated on a dry-extract basis and expressed per gram of dry extract.

### 2.6. Proximate Composition

The plant material was analyzed for moisture, dry matter, crude protein, crude fiber, crude fat, ash, and nitrogen-free extract (NFE) at the Central Scientific Research Laboratory, Trakia University (Stara Zagora, Bulgaria), which is accredited in accordance with BDS EN ISO/IEC 17025:2018 [33]. According to the official laboratory information, the laboratory applies methods established in international, regional, and Bulgarian standards in its analytical activities. The directly measured proximate-composition parameters were determined in duplicate (*n* = 2 analytical determinations) using the single pooled plant sample from each sampling point, and the results were expressed as mean ± standard deviation. Dry matter was calculated as 100 − moisture, whereas NFE was calculated by difference. All proximate-composition results were expressed as percentages.

#### 2.6.1. Determination of Moisture and Dry Matter

Moisture content was determined gravimetrically following the protocol of BDS ISO 6496:2000 [34]. Dry matter content was calculated as the difference from 100%.

#### 2.6.2. Determination of Crude Protein

Crude protein content was calculated based on the obtained values of total nitrogen content, which was determined using the Kjeldahl method according to BDS ISO 5983-1:2006 [35]. The procedure included mineralization with sulfuric acid, followed by ammonia distillation and titrimetric determination.

#### 2.6.3. Determination of Crude Fat

Crude fat content was determined according to the procedure described in BDS ISO 6492:2007 [36]. The method involves Soxhlet extraction using an organic solvent and gravimetric determination of the extracted residue.

#### 2.6.4. Determination of Nitrogen-Free Extract

Nitrogen-free extract was calculated by difference on the same percentage basis as the other proximate-composition parameters, using the following equation:

NFE (%)=100−[moisture(%)+crude protein(%)+crude fiber(%)+crude fat(%)+ash(%)]

#### 2.6.5. Determination of Crude Fiber

Crude fiber content was determined using an in-house validated method (IVM 3/2024) based on BDS EN ISO 6865:2001 [37]. The method includes sequential acid and alkaline hydrolysis with intermediate filtration, followed by washing, drying, and gravimetric determination of the remaining insoluble fibrous residue. The results were expressed as a percentage of the initial sample mass.

#### 2.6.6. Determination of Ash Content

Crude ash content was determined in accordance with ISO 5984:2022 [38] by incineration of samples until complete removal of organic matter, with the resulting mineral residue determined gravimetrically.

### 2.7. Spectrophotometric Biochemical Analyses of the Studied Plants

All spectrophotometric measurements were performed by the authors using an ONDA TOUCH UV-31 SCAN ultraviolet-visible spectrophotometer (GIORGIO BORMAC SRL, Modena, Italy). The DPPH, TPC, and TFC assays were based on the protocols described by Memdueva et al. [39].

All spectrophotometric assays were performed using freshly prepared methanolic working solutions adjusted to a dry-extract concentration of 1.0 mg/mL. For each assay, the absorbance of each independently prepared analytical sample was recorded once at each wavelength required by the corresponding method; no repeated instrumental readings of the same reaction mixture were performed.

#### 2.7.1. Determination of DPPH Radical-Scavenging Activity

DPPH radical-scavenging activity was evaluated using the 2,2-diphenyl-1-picrylhydrazyl (DPPH) radical-scavenging assay. Briefly, 100 µL of the working extract solution was mixed with 3.9 mL of 0.1 mM methanolic DPPH solution. After 30 min of incubation in the dark at room temperature, the decrease in absorbance was measured spectrophotometrically at λ = 517 nm. The results were calculated as percentage inhibition according to the standard relationship between control absorbance and sample absorbance. DPPH radical-scavenging activity was also expressed as micromoles of Trolox equivalents per gram of dry extract (µmol TE/g DE) using Trolox standard solutions for calibration over the concentration range of 5–50 µmol/L (R^2^ = 0.9991).

#### 2.7.2. Determination of Total Phenolic Content

Total phenolic content (TPC) was determined using the Folin–Ciocalteu method. A 0.5 mL aliquot of the working extract solution was mixed with 2.5 mL of Folin–Ciocalteu reagent diluted 1:10 (*v*/*v*) with deionized water. Subsequently, 2.0 mL of 7.5% (*w*/*v*) Na_2_CO_3_ solution was added. After incubation for 60 min at room temperature, the absorbance was measured spectrophotometrically at λ = 765 nm. Gallic acid was used as the calibration standard over the concentration range of 10–150 µg/mL, and the total phenolic content was calculated from the corresponding calibration curve (R^2^ = 0.9996). The results were expressed as milligrams of gallic acid equivalents per gram of dry extract (mg GAE/g DE).

#### 2.7.3. Determination of Total Flavonoid Content

Total flavonoid content (TFC) was determined using the aluminum chloride colorimetric method. Briefly, 1.0 mL of the working extract solution was mixed with 4.0 mL of deionized water and 0.3 mL of 5% (*w*/*v*) NaNO_2_ solution. After 5 min, 0.3 mL of 10% (*w*/*v*) AlCl_3_ solution was added. After an additional 6 min, 2.0 mL of 1 M NaOH was added. The mixture was homogenized, and the absorbance was measured spectrophotometrically at 510 nm. Catechin was used as the calibration standard over the concentration range of 10–150 mg/L, and the total flavonoid content was calculated from the corresponding calibration curve (R^2^ = 0.9997). The results were expressed as milligrams of catechin equivalents per gram of dry extract (mg CE/g DE).

#### 2.7.4. pH-Differential Anthocyanin Assay

Total anthocyanin content (TAntC) was determined spectrophotometrically using the pH differential method described by Lee et al. [40], with modifications. Briefly, separate 2.0 mL aliquots of each working extract solution were mixed with 8.0 mL of 0.025 M potassium chloride buffer (pH 1.0) and 8.0 mL of 0.4 M sodium acetate buffer (pH 4.5), respectively. After 20 min of incubation at room temperature, the mixtures were centrifuged at 12,000 rpm for 15 min at 4 °C. The absorbance of the supernatants was measured at λ = 520 nm and λ = 700 nm.

Total anthocyanin content was calculated according to the equation:

$$TAntC,\frac{mg}{L}=\frac{A\times MW\times D_{f}\times 1000}{\epsilon \times L}=\frac{A\times 449.2\times D_{f}}{26.9}$$

$$A={\left(A_{520}-A_{700}\right)}_{pH=1}-{\left(A_{520}-A_{700}\right)}_{pH=4.5}$$

where *A* is the corrected absorbance difference between pH 1.0 and pH 4.5; *MW* is the molecular weight of cyanidin-3-glucoside (449.2 g/mol), used as the reference basis for calculation; *Df* is the dilution factor; ϵ is the molar absorptivity of cyanidin-3-glucoside (26,900 L/mol/cm); *L* is the path length of the cuvette (1 cm); *A*_520_ and *A*_700_ are the absorbance values at 520 nm and 700 nm, respectively. The calculated assay responses, expressed as milligrams of cyanidin-3-glucoside equivalents per gram of dry extract (mg CGE/g DE), are retained in the Supplementary Data S1 for transparency.

### 2.8. Replication and Data Structure

A total of six sampling points, each representing a distinct field population, were included in the study. At each sampling point, the collected plant material was combined into one pooled sample. For each spectrophotometric assay, three analytical samples were independently prepared from the pooled plant material and analyzed separately. The directly measured proximate-composition parameters were determined in duplicate (*n* = 2 analytical determinations), whereas dry matter and NFE were calculated variables. DPPH radical-scavenging activity (expressed as both Trolox equivalents and percentage inhibition), TPC, and TFC were treated quantitatively, whereas the anthocyanin assay responses were interpreted qualitatively, as described in Section 2.7.4. The analytical samples were neither repeated measurements of the same extract nor independent biological samples. Accordingly, they quantified analytical variability only, and the resulting means and standard deviations do not estimate within-population biological variability. One corresponding soil sample was analyzed for each sampling point, providing six matched site-level soil–plant datasets. These datasets allowed an exploratory examination of covariation between plant biochemical traits and associated soil properties; however, the design did not permit the effects of species, site, collection period, and edaphic conditions to be separated.

### 2.9. Statistical Analysis

Descriptive statistics were used to summarize plant biochemical traits and soil properties at each sampling point. For moisture, crude protein, crude fiber, crude fat, and ash, point-level means and standard deviations were based on two analytical determinations (*n* = 2); dry matter and NFE were calculated variables. For DPPH radical-scavenging activity, expressed as both Trolox equivalents and percentage inhibition, TPC, and TFC, each point-level mean was based on three independently prepared analytical samples obtained from the single pooled plant sample collected at that sampling point. Accordingly, the associated standard deviations characterize analytical variability rather than within-population biological variability. The anthocyanin assay responses were interpreted qualitatively and were not included in quantitative descriptive summaries, PCA, or Spearman’s rank correlation analysis. For the descriptive summaries calculated separately for each species, arithmetic means and standard deviations were calculated across the three corresponding point-level values. These summaries were not treated as estimates of an independent species effect, and no inferential comparison between species was performed because species identity was confounded with sampling site, collection period, and associated soil conditions.

Multivariate relationships among the studied variables were examined using principal component analysis, which was applied to visualize the distribution of the six sampling points according to their biochemical and edaphic profiles. Prior to PCA, all variables were standardized (scaled to unit variance) to minimize the influence of differing measurement scales. The PCA was performed using selected plant and soil variables summarized for each sampling point. The plant variables included crude protein, ash, nitrogen-free extract, crude fat, DPPH radical-scavenging activity, percentage inhibition, total phenolic content, and total flavonoid content. The soil variables included organic matter, pH, cation exchange capacity, phosphorus, sulfur, copper, zinc, sand content, and clay content. These variables were selected to represent the principal patterns in proximate composition, DPPH radical-scavenging activity, phytochemical content, and edaphic properties within the limited set of matched sampling points.

To explore potential associations between soil properties and plant biochemical traits, Spearman’s rank correlation analysis was performed at the sampling-point level. This nonparametric approach was selected because of the small sample size and the exploratory nature of the dataset. Correlations were calculated using one matched soil dataset and one point-level plant dataset per sampling point (*n* = 6). For each soil–plant variable pair, a two-sided exact permutation *p*-value was calculated as the proportion of all 720 possible permutations that produced an absolute Spearman correlation coefficient at least as large as the absolute observed coefficient. Nominal, unadjusted *p*-values were reported, with *p* < 0.05 used as an exploratory reporting criterion rather than as confirmatory evidence. The Cu concentration at P4 was reported by the analytical laboratory as >100 ppm. For PCA and Spearman’s rank correlation analysis, this above-range result was assigned the boundary value of 100 ppm. This substitution preserved the rank order of the Cu values in Spearman’s analysis because P4 remained the highest-ranked observation; however, the contribution of Cu to the PCA was interpreted cautiously because PCA is sensitive to numerical magnitude. A heatmap of the resulting Spearman correlation matrix was prepared in Microsoft Excel (Microsoft Corporation, Redmond, WA, USA) solely to visualize the results; no statistical calculations were performed in Excel.

Descriptive analyses and PCA were performed using jamovi (version 2.7.38) [41]. The PCA implementation used the R statistical environment (version 4.5) [42] and the FactoMineR package [43,44]. Spearman’s rank correlation coefficients and their two-sided exact permutation *p*-values were calculated using R code executed through the Rj Editor within jamovi. Given the limited number of sampling points, the results of all statistical analyses were interpreted as exploratory. They were used to identify general patterns of variation and potential soil–plant associations and were not interpreted as evidence of causal relationships.

## 3. Results

The results are presented in a structured manner, including characterization of soil physicochemical properties across the six sampling sites, evaluation of proximate composition of aerial plant biomass, assessment of antioxidant and phytochemical characteristics, species-level comparison of the studied taxa, multivariate ordination based on PCA, and exploratory analysis of soil–plant associations.

### 3.1. Soil Characteristics of the Studied Populations

The six sampling points differed in their chemical and physical soil properties, confirming the edaphic heterogeneity of the studied habitats. Organic matter content ranged from the lowest value of 1.7% at P3 (*G. verum*, Banya) to the highest value of 10.3% at P5 (*G. aparine*, Tselina). Soil reaction also varied substantially, from pH 6.3 at P1 (*G. verum*, Blyagornitsa) to pH 8.1 at P4 (*G. aparine*, Banya), indicating a gradient from slightly acidic to alkaline conditions.

The soils associated with *G. aparine* were generally richer in several plant-available nutrients than those associated with *G. verum*. P ranged from 67 to 323 ppm at the *G. aparine* sites, compared with 9 to 13 ppm at the *G. verum* sites. A similar trend was observed for S, with values of 15–45 ppm in *G. aparine* sites versus 3–6 ppm in *G. verum* sites. Zn concentrations also tended to be higher in the *G. aparine* soils, reaching 175 ppm at P5, whereas the lowest value (13.7 ppm) was recorded at P2 (*G. verum*, Malka Vereya). Cu showed strong site-to-site variability, with the highest reported value at P4 (*G. aparine*), where the laboratory report indicated a concentration above 100 ppm.

Soil texture also differed among the sampling points. P2, P3, and P4 were classified as clay loam, P5 as sandy loam, and P1 and P6 as sandy silt loam. Sand content ranged from 24.3% at P2 to 55.4% at P5, whereas clay content ranged from 7.09% at P5 to 26.1% at P2. Cation exchange capacity varied between 17.5 and 28.4 meq/100 g, with the highest value recorded at P6 (*G. aparine*).

The measured soil properties varied among the sampling points in terms of organic matter status, soil reaction, nutrient availability, and texture, providing a heterogeneous edaphic background for the investigated plant populations. The selected chemical characteristics of the soils are presented in Table 2, and their microelement composition and particle-size distribution are presented in Table 3.

### 3.2. Proximate Composition of the Plant Material

The proximate composition of the aerial biomass revealed distinct variation among populations and between the two studied *Galium* species. Moisture content ranged from 6.43% at P5 (*G. aparine*) to 8.17% at P6 (*G. aparine*), while dry matter content varied from 91.8% to 93.6%, indicating low residual water content after drying.

Crude protein content was consistently higher in the *G. aparine* populations than in the *G. verum* populations. The highest value was recorded at P5 (*G. aparine*), reaching 13.0%, whereas the lowest value was observed at P2 (*G. verum*), at 9.96%. Ash content followed a similar pattern, with markedly higher values in *G. aparine* populations. The highest ash content was found at P4 *(G. aparine*), with 16.3%, while values in *G. verum* remained considerably lower, ranging from 6.85% to 7.29%. This represented the strongest compositional contrast between the two species.

At the species level, *G. verum* exhibited higher mean crude fiber, crude fat, and nitrogen-free extract contents. Crude fiber ranged from 16.2% to 18.3% in *G. verum*, compared with 12.5% to 18.0% in *G. aparine*. The highest NFE value was recorded at P2 *(G. verum*), with 55.5%, whereas the lowest value occurred at P4 (*G. aparine*), with 43.9%. Crude fat content was more variable among populations, with the highest value observed at P6 (*G. aparine*), reaching 4.42%, although similarly high values were also found at P2 and P3 (*G. verum)*.

At the species level, *G. aparine* had higher mean moisture, crude protein, and ash contents, with values of 7.46%, 12.6%, and 15.4%, respectively, compared with 6.72%, 10.3%, and 7.06% in *G. verum*. Conversely, *G. verum* was characterized by higher mean crude fiber, crude fat, and NFE contents, with values of 17.0%, 4.14%, and 54.7%, respectively, compared with 15.5%, 2.36%, and 46.7% in *G. aparine*.

These profiles show contrasts between the two sets of species-associated sampling points but do not establish an independent species effect. The point-level proximate-composition results are summarized in Table 4.

### 3.3. Phytochemical Content and DPPH Radical-Scavenging Activity

The analyses of phytochemical content and DPPH radical-scavenging activity revealed variation among the six sampled populations and between the two groups of species-associated samples. At the sampling-point level, DPPH radical-scavenging activity ranged from 13.2 ± 1.7 µmol TE/g DE at P4 (*G. aparine*) to 27.1 ± 3.5 µmol TE/g DE at P2 (*G. verum*). The percentage inhibition in the DPPH assay followed the same general pattern, with the highest point-level mean value observed at P1 (*G. verum*; 50.4 ± 2.3%) and the lowest at P4 (*G. aparine*; 20.4 ± 1.1%).

Total phenolic content (TPC) ranged from 48.6 ± 3.3 mg GAE/g DE at P1 (*G. verum*) to 65.2 ± 12.4 mg GAE/g DE at P4 (*G. aparine*). Total flavonoid content (TFC) varied within a narrower interval, from 40.6 ± 1.4 mg CE/g DE at P6 (*G. aparine*) to 49.4 ± 8.0 mg CE/g DE at P1 (*G. verum*). The pH-differential assay produced only trace-level anthocyanin responses, with generally higher signals in the *G. aparine* samples than in the *G. verum* samples. Because matrix-specific limits of detection and quantification were not established and the lowest responses showed high analytical variability, the anthocyanin results were interpreted qualitatively rather than as reliable quantitative measurements.

When the three sampling-point means were averaged for each species, the descriptive mean DPPH radical-scavenging activity was higher for the *G. verum* samples than for the *G. aparine* samples. Mean DPPH values were 26.4 µmol TE/g DE in *G. verum* and 14.7 µmol TE/g DE in *G. aparine*, while inhibition percentages were 49.0% and 21.3%, respectively. In contrast, the *G. aparine* samples showed a higher mean TPC (56.3 mg GAE/g DE) than the *G. verum* samples (51.5 mg GAE/g DE). Mean TFC was slightly higher in *G. verum* (47.7 mg CE/g DE) than in *G. aparine* (43.5 mg CE/g DE).

These results indicate variation in DPPH radical-scavenging activity and phytochemical content among the six sampled populations. The point-level dry-extract yields, DPPH radical-scavenging activity, TPC, and TFC results are summarized in Table 5.

### 3.4. Species-Level Comparison of Plant Composition

To describe species-level patterns across the sampled populations, data were aggregated by calculating the mean values of the three sampling points corresponding to each species (populations P1–P3 for *G. verum* and P4–P6 for *G. aparine*). The proximate and phytochemical datasets indicated contrasting biochemical profiles between the two *Galium* species. As summarized in Table 6, *G. verum* had higher mean DPPH radical-scavenging activity, percentage inhibition, crude fiber, crude fat, NFE, and TFC. By contrast, *G. aparine* had higher mean moisture, crude protein, ash, and TPC.

The contrast was particularly evident in DPPH radical-scavenging activity and ash content. Mean DPPH values were 26.4 µmol TE/g DE for *G. verum* and 14.7 µmol TE/g DE for *G. aparine*, while the corresponding inhibition values were 49.0% and 21.3%, respectively. Conversely, *G. aparine* had higher mean ash content than *G. verum* (15.4% and 7.06%, respectively), together with higher TPC.

The species-level descriptive comparison indicated differences in proximate composition, phytochemical content, and DPPH radical-scavenging activity between the two taxa across the studied populations.

### 3.5. Principal Component Analysis

PCA provided an integrated overview of the joint variation in plant biochemical traits and soil characteristics across the six studied populations. The first principal component (PC1) explained 62.52% of the total variance, whereas the second principal component (PC2) accounted for 21.83%. Together, PC1 and PC2 explained 84.35% of the total variance. The score plot (Figure 1a) separated the three *G. verum* sampling points from the three *G. aparine* sampling points. However, because species identity coincided with differences in sampling site, associated soil properties, and collection date, this separation describes the structure of the present dataset and cannot be interpreted as evidence of an independent taxonomic effect.

The variable loadings shown in Figure 1b and Table 7 indicated that positive PC1 scores were associated primarily with higher ash and crude protein contents and with higher values of several soil variables, including S, Cu, organic matter, Zn, pH, and P. Negative PC1 scores were associated with higher DPPH radical-scavenging activity, percentage inhibition, NFE, crude fat, clay content, and TFC. Accordingly, the *G. aparine* sampling points were positioned on the positive side of PC1, whereas the *G. verum* sampling points were positioned on its negative side. These opposing patterns describe covariation within the present dataset and do not separate species identity from sampling site, collection period, or associated soil conditions. PC2 primarily contrasted sand, P, Zn, and TFC with CEC, TPC, clay content, pH, and Cu, indicating additional site-level variation among the six sampling points.

Within the two species-associated groups, the individual sampling points did not overlap completely, with variation along PC2 being particularly evident among the *G. aparine* sampling points. This dispersion co-varied with differences in soil texture and nutrient status. The *G. verum* sampling points also showed less pronounced site-level differentiation. Because the ordination was based on only six matched site-level observations and the design contained no comparable sampling sites at which both species were collected, the PCA could not partition the relative contributions of species identity, soil properties, collection period, or collected biomass composition. The ordination should therefore be regarded as an exploratory visualization of patterns within the present dataset rather than as evidence of independent or causal effects.

### 3.6. Soil-Plant Relationships

Exploratory Spearman’s rank correlation analysis performed at the sampling-point level (*n* = 6 matched soil–plant datasets) indicated ten correlations with nominal two-sided exact permutation *p*-values below 0.05 (Table 8; Figure 2) between soil properties and plant biochemical variables. The strongest positive relationship was observed between soil Zn and crude protein (ρ = 1.000), followed by soil S and crude protein (ρ = 0.943), and soil P and crude protein (ρ = 0.943). Additional positive associations were found between soil pH and ash content (ρ = 0.899), and soil Cu and ash content (ρ = 0.886). Because the *p*-values were not adjusted for multiple comparisons and species identity was confounded with sampling site, collection period, and associated soil conditions, these relationships should be interpreted as exploratory patterns of covariation.

Among the negative correlations, the largest absolute coefficients were observed between soil P and NFE (ρ = −0.943), soil CEC and TFC (ρ = −0.943), followed by soil pH and TFC (ρ = −0.928), soil pH and percentage inhibition (ρ = −0.899), and soil Zn and NFE (ρ = −0.886). These relationships describe opposing patterns of covariation between selected soil properties and plant traits across the six matched sampling points.

Each correlation paired one composite soil dataset with the corresponding point-level plant mean derived from a single pooled plant sample. Thus, the analysis comprised six matched site-level pairs; the three independently prepared analytical samples per sampling point characterized analytical variability and did not increase the biological sample size. The correlations cannot distinguish covariation associated with species identity from that associated with sampling site, collection period, collected biomass composition, or edaphic conditions and should not be interpreted as evidence of causal relationships.

## 4. Discussion

The present dataset showed coordinated variation among species grouping, biochemical traits, and site-associated soil properties across the six sampling points. The samples from the studied populations of *G. verum* and *G. aparine* differed in several analytical traits; however, each species was represented by different sites, associated soils, and collection dates. Consequently, the observed contrasts cannot be attributed independently to taxonomic identity, edaphic conditions, seasonal variation, or differences in the composition of the collected aerial biomass. Such combined patterns are consistent with previous reports that phytochemical variation may be associated with both taxonomic and environmental factors [13,15,30,45]. Nevertheless, the present results should be interpreted as a descriptive and hypothesis-generating comparison of the studied wild populations. Across the three sampling points representing each species, the *G. verum* samples showed higher DPPH radical-scavenging activity and percentage inhibition than *G. aparine*. Although the *G. aparine* samples had higher mean TPC, their DPPH radical-scavenging activity was lower, indicating that DPPH activity was not directly proportional to TPC in the present dataset. The slightly higher mean TFC recorded in the *G. verum* samples may be relevant to this difference, as this species combined higher TFC with consistently higher DPPH radical-scavenging activity [8,11,12,14].

The higher DPPH radical-scavenging activity of *G. verum* was not accompanied by higher TPC, suggesting that the observed DPPH response may reflect qualitative differences in the extracted phytochemical fraction rather than total phenolic accumulation alone. Previous phytochemical investigations of *G. verum* have reported flavonoids, phenolic acids, iridoids, and other specialized metabolites with potential radical-scavenging activity [8,12,14]. However, individual compounds were not profiled in the present study, and their contribution to the observed DPPH response cannot be determined from the available data.

By contrast, the *G. aparine* samples were characterized by higher mean crude protein and ash contents, together with higher mean TPC. The higher ash content in the *G. aparine* samples coincided with generally greater concentrations of P, S, Zn, and Cu in their associated soils. However, because the relative proportions of plant organs were not quantified and the sampling design confounded species with site, collection period, and associated soil conditions, the observed differences in ash, crude fiber, and NFE may partly reflect variation in tissue composition, maturity, and lignification. The observed profile is consistent with the ecological tendency of *G. aparine* to occur in nutrient-enriched habitats [5]. Previous studies have also characterized the biochemical composition and bioactivity of this species [46]. Nevertheless, the present pattern should be regarded as descriptive rather than evidence of an independent species-related difference in nutrient assimilation.

Available soil P differed markedly among the sampling points, ranging from 9 to 13 ppm at the *G. verum*-associated sites and reaching 323 ppm at P5, where *G. aparine* was collected. This site-level contrast illustrates the close covariation between species grouping and edaphic conditions in the present dataset. However, because the two species were represented by different sites and collection periods, the observed pattern cannot demonstrate an independent species preference for particular soil P conditions or a causal association between soil P and the measured plant traits.

Anthocyanins are a widely distributed class of phenolic pigments in plants and were therefore included as an exploratory component of the present phytochemical assessment [47]. However, the assay responses obtained in the present study require cautious interpretation. Only trace-level signals were observed, and these were generally higher in the *G. aparine* samples than in the *G. verum* samples. Matrix-specific limits of detection and quantification were not established, and the lowest signals showed high analytical variability. The anthocyanin responses were therefore interpreted qualitatively and excluded from quantitative species-level comparisons, PCA, and Spearman’s rank correlation analysis. Consequently, no associations between anthocyanin responses and soil properties were evaluated.

The exploratory correlation analysis showed that nutrient availability and soil reaction were associated with variation in plant composition across the six sampling points. Some of these patterns, particularly those involving crude protein, ash, TFC, and percentage inhibition, are biologically plausible in light of the established roles of mineral nutrition and soil pH in plant metabolism [17,48,49,50]. However, because the analysis was based on only six matched observations, the nominal *p*-values were not adjusted for multiple comparisons, and the soil variables co-varied with species grouping, these associations are insufficient to establish direct edaphic effects and should instead be considered hypotheses for future testing.

Among these exploratory patterns, the positive correlation between soil Zn and crude protein across the six sampling points has a plausible biochemical context, as Zn is an essential micronutrient involved in numerous enzymatic and regulatory processes related to plant metabolism, including nitrogen assimilation and protein synthesis [21]. In the present dataset, the *G. aparine*-associated sampling points generally had Zn-richer soils, with soil Zn reaching 175 ppm at P5, while the corresponding plant samples also had higher crude protein contents than the *G. verum* samples. Accordingly, this correlation may reflect the shared species–site structure of the dataset rather than an independent effect of soil Zn.

These relationships should not be interpreted as simple one-factor controls. The present design aligned species identity with habitat differentiation because the *G. aparine* populations occurred on generally richer and more alkaline soils than the *G. verum* populations. Because habitat preference is itself a species-related ecological characteristic, the effects of taxonomic identity and edaphic context could not be separated in the present observational design. The correlations therefore likely reflect an integrated ecological signal rather than isolated mechanistic effects of individual soil variables. Similar associations between site conditions and metabolite accumulation have been reported in other medicinal plants [45]. This interpretation is consistent with the PCA, in which the sampling points for the two species occupied different regions of the score plot, although the analysis could not partition the respective contributions of species identity, associated soil conditions, collection period, or collected biomass composition.

Soil texture may also have been associated with these differences. The studied sites ranged from clay loam to sandy loam and sandy silt loam, implying potential differences in drainage, water availability, nutrient retention, and effective rooting conditions [51,52]. The two *G. aparine*-associated sampling points with sandier soils occupied different positions in the PCA ordination, a pattern consistent with site-level variation in relation to local soil texture. Accordingly, although the independent contribution of soil texture could not be determined, the biochemical profile at each sampling point should be interpreted in relation to both the chemical and physical properties of the associated soil rather than to nutrient concentrations alone.

An important strength of the present study is the matched soil–plant design. Each plant dataset was linked directly to a soil dataset from the same sampling point, allowing phytochemical and compositional variability to be interpreted within a specific edaphic context. This approach is consistent with studies that interpret phytochemical variation within an environmental framework [30,45]. Another strength is the integration of multiple analytical layers, including proximate composition, DPPH radical-scavenging activity, total phenolic and flavonoid contents, qualitative anthocyanin assay responses, and a relatively broad set of soil chemical and physical variables. This multidimensional approach provides a more comprehensive descriptive assessment of the studied *Galium* populations and allows their biochemical profiles to be considered in relation to the associated site conditions.

The main limitation of the study is the small number of matched field populations. With only six site-level observations, the statistical analysis necessarily remains exploratory, and the observed coefficients may partly reflect species grouping or individual site effects. An additional limitation is that all *G. aparine* populations were collected in May, whereas all *G. verum* populations were collected in June. Species identity was therefore completely confounded with collection period. Potential seasonal differences in temperature, solar radiation, plant water status, and soil nutrient availability could not be separated from the observed species-associated biochemical patterns. Although sampling was restricted to approximately the upper 30–40 cm of the shoots and roots were excluded, the relative proportions of stems, leaves, and reproductive structures were not separately quantified. Because *G. aparine* is a scrambling annual and *G. verum* is a rhizomatous perennial, the collected material may still have differed in stem-to-leaf ratio, reproductive biomass, tissue age, and degree of lignification. These differences may have contributed particularly to the observed variation in ash, crude fiber, and NFE; consequently, these compositional contrasts cannot be attributed exclusively to species-related metabolism or associated soil properties. Available soil nitrogen forms, including ammonium-N (NH_4_^+^-N) and nitrate-N (NO_3_^−^-N), were not measured, and plant biomass was not quantified. The absence of soil nitrogen data is particularly relevant to the interpretation of crude protein, which was calculated from plant total nitrogen using the Kjeldahl method. For these reasons, the observed relationships should be regarded as hypothesis-generating rather than definitive. This cautious interpretation is consistent with general methodological recommendations concerning effect-size interpretation and the limitations of small studies [53,54].

An additional analytical limitation is that the phytochemical assessment was based on bulk spectrophotometric indices, and DPPH was the only radical-scavenging assay applied. Consequently, the individual metabolites responsible for the observed responses could not be identified, and the results do not provide a comprehensive assessment of antioxidant activity. Although plant material from both species was processed using the same drying procedure, ambient drying over five days may have permitted some enzymatic oxidation of phenolic compounds and degradation of pigments. The magnitude of any such changes was not evaluated and should be considered when interpreting the observed biochemical differences. Future work should include a larger number of populations, balanced interspecific sampling across similar habitat types, repeated sampling across years and developmental stages, complementary antioxidant assays, and targeted chromatographic analyses. Such an expanded design would help distinguish species-associated patterns from environmental variation and provide a more robust basis for ecological and phytochemical generalization.

The results indicate that samples from wild *G. verum* and *G. aparine* populations in Bulgaria differ in their biochemical profiles, while species grouping co-varied with local soil conditions. The *G. verum* samples showed higher DPPH radical-scavenging activity, whereas the *G. aparine* samples showed higher ash content and TPC and were collected at sites with generally higher soil nutrient levels. Trace-level anthocyanin responses were also generally higher in *G. aparine* but were interpreted qualitatively. These findings provide a descriptive basis for considering biochemical variation among the studied wild *Galium* populations in relation to habitat context, including the associated soil properties [15,45].

## 5. Conclusions

The present study showed that the six sampled wild populations of *G. verum* and *G. aparine* differed in their biochemical profiles under the studied field conditions. Across the six sampling points, the *G. verum* samples showed higher DPPH radical-scavenging activity and percentage inhibition, whereas the *G. aparine* samples had higher mean crude protein, ash, and TPC. The anthocyanin assay responses were detectable only at trace levels and were generally higher in the *G. aparine* samples. Accordingly, these observations were interpreted qualitatively and were excluded from quantitative statistical analyses.

The matched soil–plant dataset indicated that soil chemical properties, particularly pH, P, S, Zn, Cu, and CEC, co-varied with several plant biochemical traits. Integrating these paired soil–plant analytical layers provides a valuable ecological context for the biochemical variability of wild populations. The sampling points corresponding to *G. aparine* were generally characterized by relatively nutrient-enriched and alkaline soils, whereas higher DPPH radical-scavenging activity and slightly higher mean TFC were associated with the *G. verum* samples.

Because species identity coincided with specific sampling sites, collection periods, and edaphic conditions, the descriptive results, PCA ordination, and exact-permutation Spearman correlation analysis should therefore be interpreted as exploratory and hypothesis-generating. They identify coordinated variation among the studied plant and soil characteristics but do not provide a basis for ranking the relative contributions of taxonomic and environmental factors or for establishing direct soil effects.

Future investigations should include larger population datasets, balanced sampling across comparable habitats, repeated observations across seasons or developmental stages, and complementary antioxidant assays. Broader comparisons involving additional *Galium* species would also be required before drawing conclusions about chemical variation at the genus level. Targeted analyses using high-performance liquid chromatography and liquid chromatography–mass spectrometry would allow individual phenolic acids, flavonoids, iridoids, and other metabolites to be identified and quantified and would help determine which compounds contribute to the measured DPPH radical-scavenging activity. Such studies would provide a stronger basis for distinguishing taxonomic, seasonal, and environmental sources of phytochemical variation in the two investigated species.

## Figures and Tables

**Figure 1:**
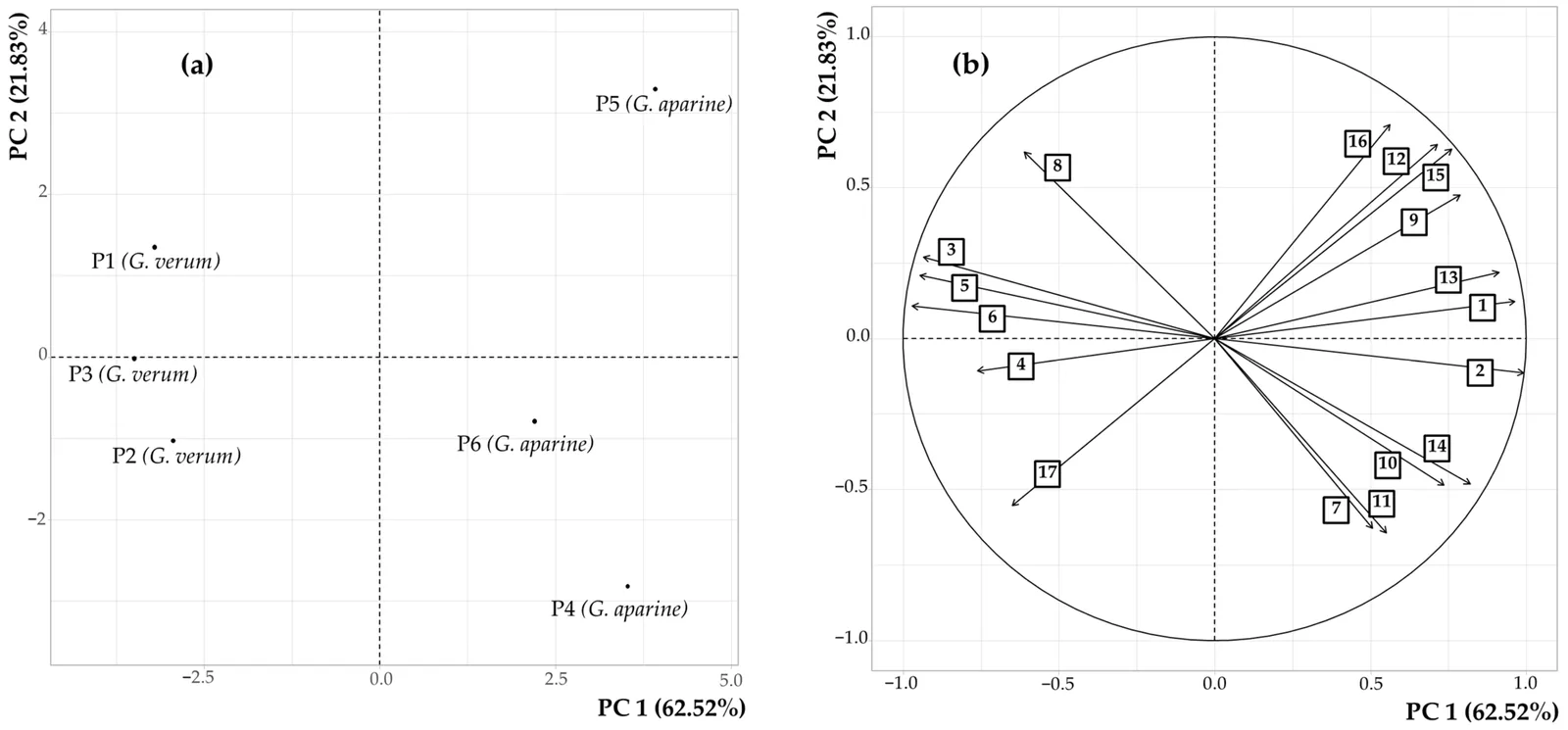
Exploratory principal component analysis (PCA) of selected plant biochemical traits and associated soil properties across six matched sampling points (*n* = 6), each representing a distinct *Galium* field population: (**a**) score plot showing the ordination of the sampling points along the first two principal components; (**b**) correlation circle showing the variable loadings on PC1 and PC2. PC1 and PC2 explained 62.52% and 21.83% of the total variance, respectively. Numbers in panel (**b**) indicate the variables included in the PCA: 1, crude protein; 2, ash; 3, nitrogen-free extract (NFE); 4, crude fat; 5, DPPH radical-scavenging activity; 6, percentage inhibition; 7, total phenolic content (TPC); 8, total flavonoid content (TFC); 9, organic matter; 10, pH; 11, cation exchange capacity (CEC); 12, phosphorus; 13, sulfur; 14, copper; 15, zinc; 16, sand; and 17, clay. The corresponding PC1 and PC2 loadings are presented in Table 7. All variables were standardized to unit variance. TAntC was excluded from the PCA because the anthocyanin assay responses were interpreted qualitatively. P1–P6, sampling points 1–6; DPPH, 2,2-diphenyl-1-picrylhydrazyl.

**Figure 2:**
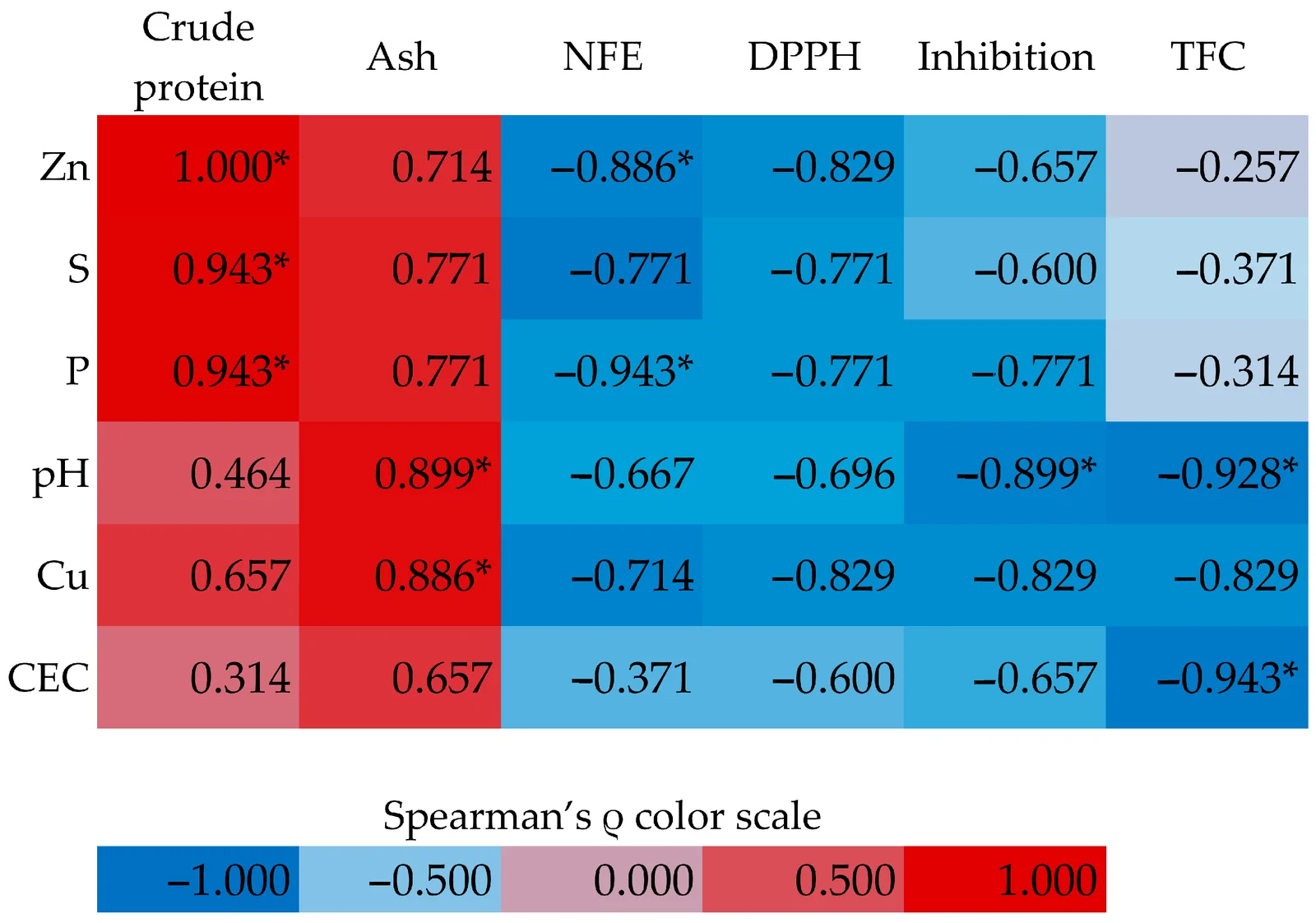
Heatmap of sampling-point-level Spearman’s rank correlations between selected soil properties and plant traits, based on six matched soil–plant datasets (*n* = 6). Cell values represent Spearman’s ρ; red indicates positive and blue indicates negative correlations. Asterisks indicate associations with nominal two-sided exact permutation *p* < 0.05, calculated by complete enumeration of all 720 possible permutations. The *p*-values were not adjusted for multiple comparisons. The Cu concentration reported as >100 ppm at P4 was assigned the boundary value of 100 ppm. CEC, cation exchange capacity; Cu, copper; DPPH, 2,2-diphenyl-1-picrylhydrazyl radical-scavenging activity; NFE, nitrogen-free extract; P, phosphorus; S, sulfur; TFC, total flavonoid content; Zn, zinc.

**Table 1:** Sampling points, floristic affiliation, and locality of the studied *Galium* populations.

Sampling Point	Species	Locality	Geographic Coordinates	Altitude (m a.s.l.)	Floristic Region
Point 1 (P1)	*G. verum*	Blyagornitsa	42°41′10.9″ N 25°55′52.7″ E	396	Eastern Stara Planina
Point 2 (P2)	*G. verum*	Malka Vereya	42°24′28.6″ N 25°32′14.3″ E	353	Thracian Lowland
Point 3 (P3)	*G. verum*	Banya	42°36′19.0″ N 25°59′59.0″ E	231	Tundzha Hilly Plain
Point 4 (P4)	*G. aparine*	Banya	42°36′05.4″ N 26°00′10.9″ E	231	Tundzha Hilly Plain
Point 5 (P5)	*G. aparine*	Tselina	42°07′33.7″ N 25°26′43.1″ E	139	Thracian Lowland
Point 6 (P6)	*G. aparine*	Blyagornitsa	42°41′11.3″ N 25°56′10.2″ E	396	Eastern Stara Planina

Note: a.s.l., above sea level.

**Table 2:** Selected chemical characteristics of the soils associated with the studied populations.

Sampling Point	OM (%)	pH	CEC (meq/100 g)	Ca (ppm)	Mg (ppm)	K (ppm)	P (ppm)	S (ppm)	Na (ppm)
P1	3.5	6.3	17.8	2322	415	133	10	6	35
P2	4.7	7.6	21.6	4542	280	280	9	5	17
P3	1.7	7.0	17.5	2006	585	123	13	3	40
P4	5.1	8.1	25.4	5293	291	444	68	35	38
P5	10.3	7.6	18.8	3079	537	1000	323	45	31
P6	6.2	8.0	28.4	4785	282	821	67	15	57

Note: P1–P6, sampling points 1–6; OM, organic matter; CEC, cation exchange capacity; Ca, calcium; Mg, magnesium; K, potassium; P, phosphorus; S, sulfur; Na, sodium.

**Table 3:** Microelement composition and particle-size distribution of the studied soils.

Sampling Point	B (ppm)	Cu (ppm)	Mn (ppm)	Mo (ppm)	Fe (ppm)	Zn (ppm)	Sand (%)	Silt (%)	Clay (%)	Texture Class
P1	1.42	8.9	58	0.08	464	39.5	34.7	49.0	16.3	Sandy Silt Loam
P2	1.81	11.1	336	0.04	225	13.7	24.3	49.6	26.1	Clay Loam
P3	0.84	3.6	88	0.07	298	31.7	30.2	44.8	25.0	Clay Loam
P4	4.64	>100	161	0.08	61	65.5	26.1	52.6	21.3	Clay Loam
P5	10.3	39.8	63	0.05	384	175	55.4	37.5	7.09	Sandy Loam
P6	8.77	43.6	201	0.04	238	64.1	47.3	44.5	8.21	Sandy Silt Loam

Note: P1–P6, sampling points 1–6; B, boron; Cu, copper; Mn, manganese; Mo, molybdenum; Fe, iron; Zn, zinc. The Cu concentration at P4 was reported by the analytical laboratory as >100 ppm.

**Table 4:** Proximate composition of the aerial parts of the studied *Galium* populations.

Sampling Point	Moisture (%)	Dry Matter (%)	Crude Protein (%)	Crude Fiber (%)	Crude Fat (%)	Ash (%)	NFE (%)
P1	6.64 ± 0.09	93.4	10.9 ± 0.41	16.6 ± 0.16	3.92 ± 0.07	6.85 ± 0.10	55.1
P2	6.90 ± 0.06	93.1	9.96 ± 0.27	16.2 ± 0.16	4.13 ± 0.08	7.29 ± 0.18	55.5
P3	6.61 ± 0.10	93.4	10.0 ± 0.23	18.3 ± 0.38	4.38 ± 0.09	7.04 ± 0.08	53.7
P4	7.79 ± 0.13	92.2	12.7 ± 0.23	18.0 ± 0.22	1.31 ± 0.06	16.3 ± 0.12	43.9
P5	6.43 ± 0.08	93.6	13.0 ± 0.14	15.9 ± 0.15	1.36 ± 0.08	15.3 ± 0.28	48.0
P6	8.17 ± 0.20	91.8	12.1 ± 0.20	12.5 ± 0.13	4.42 ± 0.12	14.7 ± 0.21	48.1

Note: Values for moisture, crude protein, crude fiber, crude fat, and ash are means ± standard deviations of two analytical determinations (*n* = 2) performed on one pooled plant sample from each sampling point. These analytical replicates characterize laboratory variability and do not represent biological replication or within-population variability. Dry matter was calculated as 100 − moisture. Nitrogen-free extract (NFE) was calculated as dry matter − (crude protein + crude fiber + crude fat + ash). P1–P6, sampling points 1–6; NFE, nitrogen-free extract.

**Table 5:** Dry-extract yield, phytochemical content and DPPH radical-scavenging activity of the studied *Galium* populations (mean ± SD, *n* = 3).

Sampling Point	Dry-Extract Yield (%)	DPPH (µmol TE/g DE)	Inhibition (%)	TPC (mg GAE/g DE)	TFC (mg CE/g DE)
P1	29.3 ± 3.2	25.9 ± 1.6	50.4 ± 2.3	48.6 ± 3.3	49.4 ± 8.0
P2	27.9 ± 1.9	27.1 ± 3.5	50.3 ± 3.1	54.5 ± 1.6	45.9 ± 3.5
P3	26.4 ± 3.1	26.1 ± 1.2	46.2 ± 4.6	51.2 ± 2.7	47.9 ± 2.4
P4	22.2 ± 1.5	13.2 ± 1.7	20.4 ± 1.1	65.2 ± 12.4	42.8 ± 4.8
P5	19.4 ± 0.6	17.0 ± 1.1	22.8 ± 0.8	53.2 ± 5.2	47.1 ± 8.2
P6	21.6 ± 1.0	13.8 ± 1.1	20.8 ± 0.8	50.4 ± 1.9	40.6 ± 1.4

Note: Values are means ± standard deviation (SD) of three independently prepared analytical samples from one pooled plant sample per sampling point (*n* = 3). These analytical replicates do not represent biological replication or within-population variability. Dry-extract yield was determined gravimetrically and is expressed as a percentage of the initial dry plant mass. P1–P6, sampling points 1–6; SD, standard deviation; DPPH, 2,2-diphenyl-1-picrylhydrazyl; TE, Trolox equivalents; DE, dry extract; TPC, total phenolic content; GAE, gallic acid equivalents; TFC, total flavonoid content; CE, catechin equivalents.

**Table 6:** Descriptive summary of proximate composition, DPPH radical-scavenging activity, and phytochemical characteristics of the sampled *G. verum* and *G. aparine* populations, based on sampling-point means.

Species	Moisture (%)	Crude Protein (%)	Crude Fiber (%)	Crude Fat (%)	Ash (%)	NFE (%)	DPPH (µmol TE/g DE)	Inhibition (%)	TPC (mg GAE/g DE)	TFC (mg CE/g DE)
*G. aparine*	7.46 ± 0.91	12.6 ± 0.42	15.5 ± 2.75	2.36 ± 1.78	15.4 ± 0.84	46.7 ± 2.41	14.7 ± 2.01	21.3 ± 1.33	56.3 ± 7.87	43.5 ± 3.33
*G. verum*	6.72 ± 0.16	10.3 ± 0.54	17.0 ± 1.10	4.14 ± 0.23	7.06 ± 0.22	54.7 ± 0.95	26.4 ± 0.68	49.0 ± 2.43	51.5 ± 2.96	47.7 ± 1.75

Note: Values are means ± standard deviations calculated across the three sampling-point means for each species (*n* = 3 sampling points per species). These values are descriptive summaries, and the SD represents variation among the sampling points rather than within-population biological variability. No inferential comparison between species was performed. NFE, nitrogen-free extract; DPPH, 2,2-diphenyl-1-picrylhydrazyl; TE, Trolox equivalents; DE, dry extract; TPC, total phenolic content; GAE, gallic acid equivalents; TFC, total flavonoid content; CE, catechin equivalents.

**Table 7:** Variable coordinates (correlation loadings) on the first two principal components of the exploratory principal component analysis.

No	Variable	PC1	PC2
1	Crude protein	0.964	0.122
2	Ash	0.991	−0.114
3	Nitrogen-free extract (NFE)	−0.934	0.269
4	Crude fat	−0.761	−0.108
5	DPPH radical-scavenging activity	−0.946	0.209
6	% Inhibition	−0.970	0.107
7	Total phenolic content (TPC)	0.507	−0.628
8	Total flavonoid content (TFC)	−0.610	0.617
9	Organic matter	0.788	0.474
10	pH	0.737	−0.485
11	Cation exchange capacity (CEC)	0.550	−0.644
12	Phosphorus	0.714	0.643
13	Sulfur	0.912	0.219
14	Copper	0.820	−0.482
15	Zinc	0.760	0.628
16	Sand	0.563	0.709
17	Clay	−0.650	−0.553

Note: The PCA was based on six matched site-level soil–plant datasets (*n* = 6), with all variables standardized to unit variance. PC1 and PC2 explained 62.52% and 21.83% of the total variance, respectively, accounting for 84.35% cumulatively. TAntC was excluded from the PCA because the anthocyanin assay responses were interpreted qualitatively. DPPH, 2,2-diphenyl-1-picrylhydrazyl; NFE, nitrogen-free extract; TPC, total phenolic content; TFC, total flavonoid content; CEC, cation exchange capacity.

**Table 8:** Exploratory sampling-point-level Spearman correlations with nominal two-sided exact permutation *p*-values below 0.05.

Soil Variable	Plant Variable	Spearman’s ρ	*p*-Value	Direction
Zn	Crude protein	1.000	0.0028	Positive
S	Crude protein	0.943	0.0167	Positive
P	Crude protein	0.943	0.0167	Positive
pH	Ash	0.899	0.0278	Positive
Cu	Ash	0.886	0.0333	Positive
CEC	TFC	−0.943	0.0167	Negative
P	NFE	−0.943	0.0167	Negative
pH	TFC	−0.928	0.0167	Negative
pH	Inhibition	−0.899	0.0278	Negative
Zn	NFE	−0.886	0.0333	Negative

Note: Spearman’s ρ was calculated from one matched soil dataset and one point-level plant dataset per sampling point (*n* = 6). Two-sided exact permutation *p*-values were obtained by complete enumeration of all 720 possible permutations. The *p*-values were not adjusted for multiple comparisons. The Cu concentration at P4, reported as >100 ppm, was assigned the boundary value of 100 ppm. Because species identity was confounded with sampling site, collection period, and associated soil conditions, all correlations should be interpreted as exploratory. CEC, cation exchange capacity; Cu, copper; NFE, nitrogen-free extract; P, phosphorus; S, sulfur; TFC, total flavonoid content; Zn, zinc.

## Data Availability

The data presented in this study are available in the Supplementary Materials. Additional information is available from the corresponding author upon reasonable request.

## Supplementary Materials

The following supporting information can be downloaded at: https://www.mdpi.com/article/10.3390/plants15192917/s1. Supplementary Data S1: Supporting data for six wild populations of *G. verum* L. and *G. aparine* L., including sampling-site information, plant proximate composition, raw biochemical data, soil properties, and statistical input data for PCA and Spearman’s rank correlation analysis.

## Abbreviations

The following abbreviations are used in this manuscript:

CE	Catechin Equivalents
CEC	Cation Exchange Capacity
CGE	Cyanidin-3-Glucoside Equivalents
DE	Dry Extract
DPPH	2,2-Diphenyl-1-Picrylhydrazyl
EDTA	Ethylenediaminetetraacetate
GAE	Gallic Acid Equivalents
ICP	Inductively Coupled Plasma
LOI	Loss On Ignition
NFE	Nitrogen-Free Extract
OM	Organic Matter
PC1	First Principal Component
PC2	Second Principal Component
PCA	Principal Component Analysis
SD	Standard Deviation
TAntC	Total Anthocyanin Content
TE	Trolox Equivalents
TFC	Total Flavonoid Content
TPC	Total Phenolic Content
UKAS	United Kingdom Accreditation Service
